# Identification of Cellular Targets of MicroRNA-181a in HepG2 Cells: A New Approach for Functional Analysis of MicroRNAs

**DOI:** 10.1371/journal.pone.0123167

**Published:** 2015-04-22

**Authors:** Jane Yi Lin Tan, Nagy A. Habib, York Wieo Chuah, Yin Hoe Yau, Susana Geifman-Shochat, Wei Ning Chen

**Affiliations:** 1 School of Chemical and Biomedical Engineering, College of Engineering, Nanyang Technological University, Singapore, Singapore; 2 Imperial College London, Faculty of Medicine, Department of Surgery and Cancer, London, England; 3 Division of Structural Biology and Biochemistry, School of Biological Sciences, Nanyang Technological University, Singapore, Singapore; H.Lee Moffitt Cancer Center & Research Institute, UNITED STATES

## Abstract

MicroRNAs (miRNAs) are known to play a part in regulating important cellular processes. They generally perform their regulatory function through their binding with mRNAs, ultimately leading to a repression of target protein expression levels. However, their roles in cellular processes are poorly understood due to the limited understanding of their specific cellular targets. Aberrant levels of miRNAs have been found in hepatocellular carcinoma (HCC) including miR-181a. Using bioinformatics analysis, cyclin-dependent kinase inhibitor 1B (CDKN1β) and transcriptional factor E2F7 were identified as potential targets of miR-181a. Validation analysis using surface plasmon resonance (SPR) showed a positive binding between miR-181a and the 3’UTRs of these two potential mRNA targets. *In vivo* luciferase assay further confirmed the positive miR-181a:mRNA bindings, where a significant decrease in luciferase activity was detected when HepG2 cells were co-transfected with the 3’UTR-containing reporter plasmids and miR-181a. The potential impact of miR-181a binding to its specific targets on the general cellular behavior was further investigated. Results showed that miR-181a significantly activated the MAPK/JNK pathway which regulates cell proliferation, supporting our recently reported findings. Inhibition of miR-181a, on the other hand, abolished the observed activation. Our findings open up a new approach in designing targeted functional analysis of miRNAs in cellular processes, through the identification of their cellular targets.

## Introduction

Cancer is one of the top leading causes of mortality in the world today, accounting for approximately 7.6 million deaths in 2008 [1]. Its progression is usually a multistep process, involving complex genetic changes that gradually transform a cell from a pre-malignant state into invasive cancer and finally metastasis. What makes cancer cells so resilient to current therapies is its ability to proliferate in an autonomous manner, avoid inhibitory growth signals, escape apoptosis pathways, stimulate and sustain angiogenesis and spread to other parts of the body. Often, genes implicated in cell proliferation, differentiation and apoptosis are involved in cancer initiation and subsequent progression [2].

Apart from the classic protein coding genes, the recent discovery of miRNAs has also highlighted the roles they play in the development of cancer. MiRNAs form a subclass of small RNAs and are single-stranded, non-coding RNAs of 19–25 nucleotides in length, and are usually found in the intergenic regions or introns of the genome [3]. They are able to regulate various physiologically important processes in the body such as cellular proliferation, differentiation, cell cycle, angiogenesis, metabolism, immune response and apoptosis, making them a key player in cancer [4]. Studies have shown that different miRNA expression profiles are often seen associated with the different stages of cancer. Like any gene, miRNAs (in a specific tissue and/or differentiation state) may act as an oncogene or a tumor suppressor, depending on the downstream effect in has in the cell. MiRNAs may post-transcriptionally regulate gene expression by either cleavage or translational repression of mRNA through binding to the 3′-untranslated region (3′-UTR) of the target genes [3]. MiRNAs are able to regulate different cellular processes because a single miRNA is able to negatively regulate multiple target proteins through direct interaction with the corresponding mRNAs. Approximately 3% of the entire human genome encodes for miRNAs and they regulate up to 30% of human protein coding genes [5]. Because they are able to regulate a large number of proteins, their abnormal expression often disrupts the functioning of the cell, either by activating oncogenes or deactivating tumor suppressor networks. This contributes to the initiation and progression of many human cancers.

Although the importance of miRNAs can be seen through the myriad of cellular functions they are involved in, a lot is still unknown about their mechanism of action and gene targets. In this study, we investigate the involvement of miR-181a in HCC, the third leading cause of cancer-related deaths, and its potential mRNA binding targets in HepG2 cells. MiR-181a is not only found to be upregulated in HCC but also in Hepatic Stem Cell-like HCC (HpSC-HCC) [6]. These are HCC cells that are EpCAM and AFP positive (i.e. EpCAM^+^AFP^+^ HCC), which respectively, serves as a hepatic stem/progenitor cell-specific marker and a marker indicative for HCC [6]. MiR-181a is also involved in the activated Wnt/β-catenin signaling pathway in HpSC-HCC [6]. This pathway has been found to be over-activated in at least 60% of HCC, as the levels of β-catenin protein in the nucleus and/or cytoplasm was found to be increased in these cases. Our previous study [7] on the overall effect miR-181a has on HepG2 cells showed a significant increase in cell viability with the transfection of miR-181a. The opposite was observed when miR-181a was inhibited, where cell viability dropped by 20%. Inhibiting miR-181a also delayed HepG2 cell cycle progression to an extent more than miR-181a encouraged cell cycle entry. These results may suggest that miR-181a enhances liver cancer cell growth while inhibiting it represses proliferation and cell cycle. Its mechanism of action and mRNA targets will be further studied here.

The study of miRNA:mRNA interaction and binding will be carried out both under *in vitro* and *in vivo* conditions. Surface plasmon resonance (SPR) is a real-time platform for detecting the binding of specific molecules to a partner. Protein interactions, small molecules, nucleic acids, cells and viruses and carbohydrates are some of the compounds that can be studied with SPR [8]. It typically takes place on the sensor surface of a chip, and its working principle is based on the change in refractive index of the interface when the surface condition changes. When positive binding occurs, the sensorgram registers a change in angle due to the increase in mass bound at the surface. A real-time profile of binding followed by subsequent dissociation can be generated, and the kinetics of the binding characterized. This provides a quick yet precise method of screening potential mRNA targets of miR-181a. Following successful identification of possible targets *in vitro*, *in vivo* experiments via luciferase assays were performed to validate the binding interactions within the cellular environment.

## Materials and Methods

### Cell Culture

HepG2 cells (American Type Culture Collection) were maintained in monolayer culture in Minimal Essential Medium Eagle (MEM) (Gibco, Invitrogen), supplemented with 10% FBS in a 5% CO_2_ incubator at 37°C. Subculturing of cells was carried out using 0.25% trypsin-EDTA (Life Technologies, Invitrogen).

### Bioinformatics Study of mRNA Target Prediction

Human miRNA target predictions for miRNA families were obtained from TargetScan 6.2 (http://www.targetscan.org/) as well as miRanda (http://www.microrna.org/microrna/home.do) database. miRò, the miR-ontology database was also used as a convenient source of categorizing miRNAs and their targets by diseases, functions or processes that they are involved in. Relevant targets predicted by both TargetScan and miRanda were chosen for wet lab experimentation. Putative miRNA:mRNA interaction, in TargetScan, was based on the total context score and probability of conserved targeting (P_CT_). The more negative the context score and the higher the P_CT_, the higher the probability of miRNA:mRNA binding.

### Surface Plasmon Resonance (SPR) assay

All SPR experiments were run with HBS-EP buffer (10 mM HEPES, 150 mM NaCl, 3.4 mM EDTA and 0.005% P20 at pH 7.4) on a Biacore 3000 (BIAcore AB, GE Healthcare) with a carboxymethylated dextran coated sensor chip (CM5) at 25°C. Two surfaces were activated for 7 min with 1:1 mixture of 0.2 M N-ethyl-N’-[3-(diethylamino)propyl]carbodiimide (EDC) and 50 mM N-hydroxysuccinimide (NHS), before immobilization of 1500 RU of Neutravidin (Pierce, USA) in 10 mM sodium acetate at pH 6.0 at the flow rate of 10 μl/min by standard amine coupling procedure. The surfaces were then blocked with 0.5 M ethanolamine-HCl at pH 8.5 for 7 min. Biotin-labeled single-stranded RNA harboring 31 bp 3’UTR of CDKN1β mRNA (5’- GGGAGUU**UUGAAUGU**UAAGAAUUGACCAUCUGC -3’) and 34 bp 3’UTR of E2F7 mRNA (5’- GGGUAUGACGAC**UUGAAUGU**UUAUACUUUUAUUC -3’) were captured on sensor chip surface to 200 RU and 430 RU, respectively. Another two empty channels serve as reference. A 2-fold serial injection of miR-181a (180 nM, 359 nM, 719 nM, 1.44 μM, 2.88 μM, 5.75 μM, 11.5 μM, and 23 μM) was injected at 10 μL/min across all the surfaces for 2 min, and was then allowed to dissociate for 15 min. All the sensorgrams were corrected by subtraction of the responses of analytes on the empty channel and buffer blanks [9]. Processed data were globally analyzed and fit into 1:1 interaction model to yield the affinity.

### Transformation

Mammalian expression vectors (Firefly/renilla duo-reporter vector system) with the 3’ UTR of CDKN1β and E2F7 cloned downstream of the secreted firefly luciferase reporter gene were obtained from GeneCopoeia. These have been sequenced and quality checked before subsequent usage. 1μl of DNA plasmid was mixed with 50 μl competent cell *Ecoli* strain *TOP10* in a microcentrifuge tube. The tube was stored on ice for 10min. It was next heat shocked at 42°C for 90s and returned to ice immediately for 2min. 450μl fresh LB was added and the cells incubated at 37°C in a shaking incubator at 250 rpm for 1 h. LB/*kanamycin* agar plate (1% Tryptone, 0.5% yeast extract, 171mM NaCl, 1.5% agar, PH 7.0) was prepared and 100μl of transformed bacterial cells was spread on top and incubated at 37°C overnight in an inverted position.

### Mini-prep Purification of Plasmids

A single colony was incubated in 5ml sterile LB medium with 100ng/ml *Kanamycin* in a 15 ml falcon tube at 37°C with agitation at 250 rpm overnight. Bacteria were harvested directly by centrifuging at 2500 rpm for 15min. Plasmid extraction was carried out using QIAprep Miniprep plasmid kit (Qiagen) according to the manufacturer’s instructions. The eluted DNA was quantified by a nanodrop spectrophotometer and stored at -20°C until further use.

### Co-Transfection of Plasmids and miRNAs

The co-transfection of DNA plasmids and miRNAs was carried out via electroporation. Briefly, 1x10^6^ of overnight starved HepG2 cells were resuspended in 100ul nucleofection solution and co-transfected with 4ug DNA plasmid and either 10nM or 100nM miR-181a. An additional control vector was also used such that the sequence cloned downstream of the firefly luciferase reporter gene is of a random, non-specific targeting sequence. 100K transfected cells were seeded in each well of a 24-well plate and incubated with 500ul complete growth media at 37°C overnight.

### Luciferase Assay

The luciferase assay was carried out using GeneCopoeia Luc-Pair miR Luciferase Assay kit. 24h after co-transfection of plasmids and miRNAs, growth media was aspirated and 300μl Solution 1 was added to each well and incubated at room temperature for 3 minutes. 80μl of cell lysate was removed and transferred into a new 1.5ml microcentrifuge tube. Working Solution I and Working Solution II are prepared according to the manufacturer’s instructions. Prior to luciferase activity measurement, the GloMax 20/20 Luminometer (Promega) was set to measure luminescence for 2s. 20μl of Working Solution I was added to each of the 80μl sample and firefly luminescence measured (M1). 100μl of Working Solution II was subsequently added and renilla luminescence measured (M2). The values obtained were normalized by taking M1/M2.

### Western Blot

SDS-PAGE was performed on the Bio-Rad mini-protean electrophoresis system. 30μg of total protein was extracted per sample and mixed with 6 X SDS sample buffer and heated at 90°C for 5 min. Novex Sharp Protein Standard (Life technologies, Invitrogen) was used to verify protein size. Proteins were transferred onto Polyvinylidene Fluoride (PVDF) membrane and subjected to blocking in 5% non-fat milk in 1 X PBS for 1h at room temperature. Following blocking, the membrane was allowed to incubate with the respective primary antibodies in 5% non-fat milk / 1 X PBS solution overnight at 4°C. The membrane was washed with 0.5% PBST 3 times before incubating with secondary antibody tagged with Horseradish Peroxidase (HRP) in 5% non-fat milk / 1 X PBS solution for 1h. The membrane was washed again another 3 times and incubated in chemiluminescence (ECL) using SuperSignal West Pico chemiluminescent substrate (Pierce). The antibodies used in this study were as follows: (1) p27 (SC 528, Santa Cruz), (2) E2F7 (AB56022, Abcam, Biomed Diagnostics), (3) *β*-actin mouse monoclonal IgG (Sigma A5441).

### Cignal Reporter Assay Analysis of Cancer Pathways Affected by miR-181a

The Cancer 10-pathway Reporter Luciferase Kit (Qiagen) in plate format was used in the study of ten cancer-related signaling pathways. Briefly, 2pmol of miRNA was diluted in 25μl of pure Opti-MEM (Life Technologies) growth media and added to each well of the plate to resuspend the plasmids. 0.6μl Attractene (Qiagen) was diluted in 25μl pure Opti-MEM in a separate microcentrifuge tube, incubated at room temperature for 5min and added to each well of the resuspended plasmids. The Attractene/plasmids-miRNA complex was allowed to form for 20min at room temperature. HepG2 cells growing in complete MEM media with 10% FBS were washed in PBS and trypsinized, centrifuged and supernatant discarded. The cell pellet was resuspended to 4X10^5^ cells/ml in Opti-MEM containing 5% FBS. After 20 minutes of complex formation, 100μl of the prepared cell suspension was added to each well containing the constructs-miRNA-Attractene complexes, making up to a final volume of 150μl per well of a 96-well plate. The plate was mixed gently with a rocking motion and then incubated at 37°C in a 5% CO_2_ incubator for 16-24h. After 16-24h of transfection, the media was aspirated and changed to 75μl complete growth media (MEM with 10% FBS and 1% antimycotics) and further incubated at 37°C for another 24h. The cells were checked with the positive control for GFP fluorescence using a fluorescent microscope. Following successful transfection validation, the luciferase assay was developed by using Dual-Luciferase Reporter Assay System (Promega).

### Developing Luciferase (Plate) Assay

Briefly, the Dual-Glo Luciferase buffer was added to Dual-Glo Luciferase Substrate to make up the Dual-Glo Luciferase Reagent. A calculated amount of Dual-Glo Stop & Glo Reagent was prepared by diluting the Dual-Glo Stop & Glo Substrate to the Dual-Glo Stop & Glo Buffer in a ratio of 1:100. 75μl Dual-Glo Luciferase Reagent was added to each well with a multichannel pipette and firefly luminescence was measured after 10min (M1). 75ul of Dual-Glo Stop & Glo Reagent was added to each well and the renilla luminescence measured after 10min (M2). The ratio of luminescence (M1/M2) of firefly to renilla gives the normalized luminescent values per well. Luminescence was measured in a Tecan microplate reader with a Magellan Data Analysis Software.

### RNA Extraction and Quantification

RNA isolation was carried out using RNeasy mini kit (Qiagen). Briefly, cell pellets were resuspended in 350ul buffer RTL. (10ul β-Mercaptoethanol (β-ME) was added to 1ml buffer RTL). A 20-gange needle (0.9mm diameter) fitted to an RNase-free syringe was used to homogenize the sample, 1 volume 70% ethanol added and mixed well. Samples were transferred to RNeasy mini column placed in a 2ml collection tube, centrifuged and washed repeatedly and finally eluted with RNase-free water. The flow-through containing total RNA was quantified with a nanodrop spectrophotometer and stored at -80°C until further use.

### Reverse Transcription

Reverse transcription was carried out using the RT^2^ First Strand Kit (Qiagen). 0.5ug total RNA was added to the genomic DNA elimination mix, incubated at 42°C for 5min and placed on ice. 10ul of reverse-transcription mix was added to 10ul of genomic DNA elimination mix, pipetted to mix and incubated at 42°C for 15min, followed by a 95°C incubation for 5min. 91ul RNase-free water was added per reaction and the mixture either stored at 20°C or immediately used for real-time PCR.

### Real Time PCR

25ul of the PCR mix was added to each well of the custom made RT^2^ Profiler PCR Array using an 8-channel pipettor. The array was sealed with optical thin-wall 8-caps strips. The platform used to carry out real-time PCR was Applied Biosystems 7500, with the following cycling conditions: 95°C for 10min (1 cycle); 95°C for 15s, 60°C for 1min (40 cycles). An automated baseline was used and the following calculation was used in the analysis of results: Sample ΔC_t_ = C_t_ Sample-C_t_ Housekeeping gene; ΔΔC_t_ = Sample ΔC_t_-Control ΔC_t_; Fold change = 2 –ΔΔC_t_

### Statistical analysis

All data are presented as mean ± SD. A 2-tailed Student’s *t*-test was used for the statistical analysis of raw data obtained. Statistical significance was accepted at *p* < 0.05.

## Results and Discussion

### Bioinformatics Screening of Putative mRNA Targets

**Fig 1:**
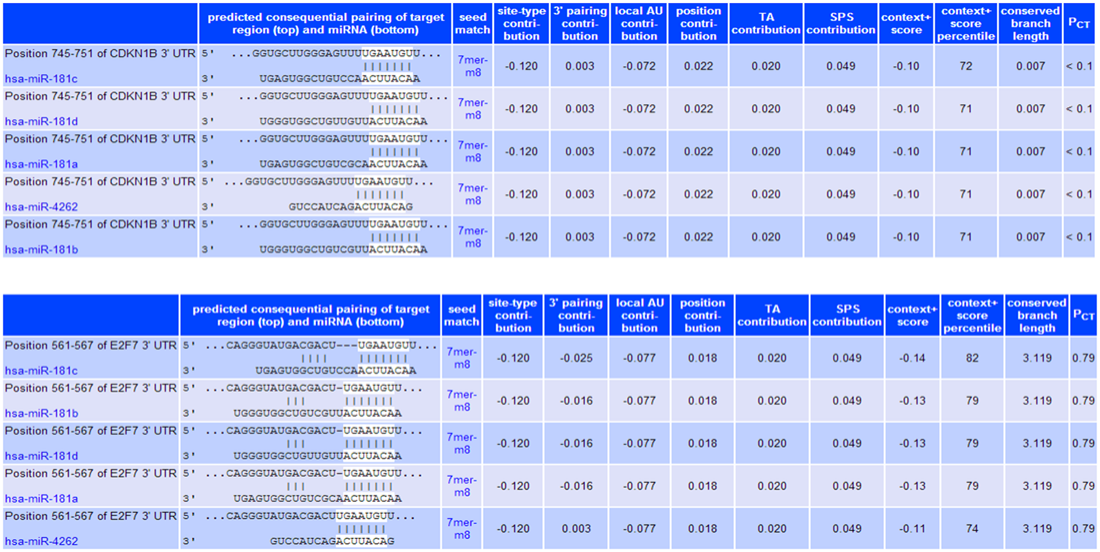
Targetscan’s [10] predicted binding sites of miR-181a to the 3’UTRs of CDKN1β and E2F7. The total context score is based on six features: site-type contribution, 3' pairing contribution, local AU contribution, position contribution, TA (target site abundance) contribution and SPS (seed-pairing stability) contribution. The more negative the context score, the higher the probability of mRNA binding. The probability of conserved targeting, P_CT_, refers to the likelihood of the sequence being conserved so as to allow regulation by the miRNA. The higher the P_CT_, the higher the chance of miRNA:mRNA binding.

Three online software and databases, namely, TargetScan 6.2, miRanda and miRò, were used in the first step to elucidate potential mRNA targets of miR-181a. Putative targets that were predicted in at least two of the software were chosen for further study. Additionally, among the many possible targets predicted and listed, those that are involved in important cancer-related processes were selected for subsequent wet lab analysis. Two such targets of interest are cyclin-dependent kinase inhibitor 1B (CDKN1β) and transcription factor E2F7 (E2F7). According to TargetScan 6.2, the more negative the context score of an interaction, the more favorable it is for binding to occur. The context score is based on six types of interactions found both at the seed region and around the seed region. Fig 1 shows the putative binding sites and binding scores of miR-181 family to the 3’ UTRs of CDKN1β and E2F7. It seems that there is a more extensive base pairing between miR-181a and E2F7 as compared to CDKN1β due to the presence of predicted base interactions beyond the seed region, leading to a more negative context score. Using this information as the first step, we proceeded to test for actual binding between the two RNAs.

### *In vitro* Binding of miR-181a to the 3’UTR of CDKN1β and E2F7

**Fig 2:**
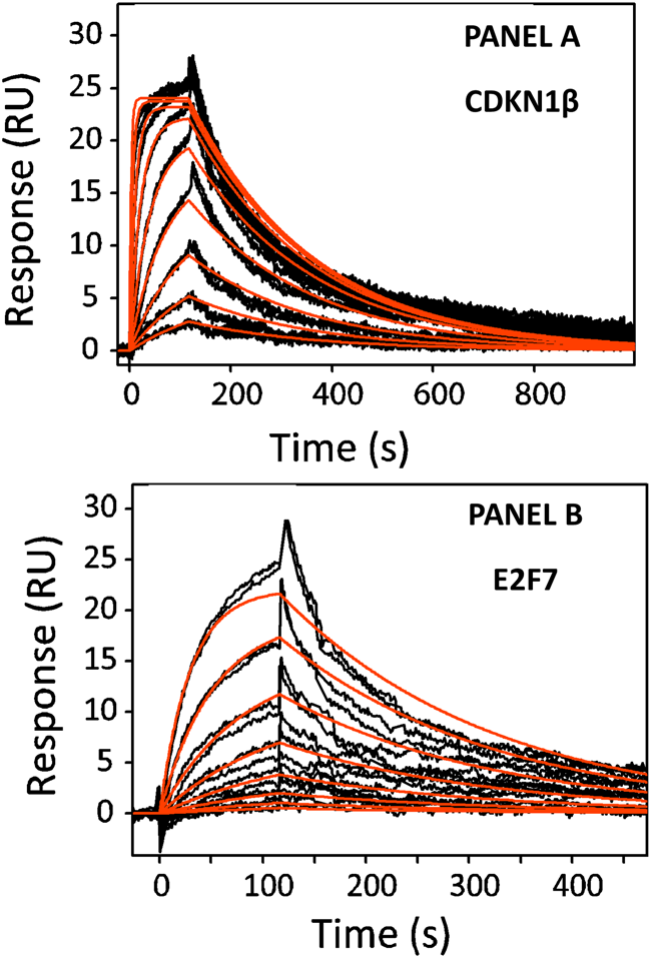
Experimental curves of the bindings between miR-181a and CDKN1β and E2F7 (Panels A and B). A positive 1:1 binding is observed between both miR-181a and CDKN1β and E2F7 with dissociation constants 272.5 ± 0.008 nM and 1.186 ± 0.009 uM respectively.

Based on the results of the bioinformatics analysis, we proceeded to use surface plasmon resonance (SPR) to monitor miR-181a:mRNA interactions, if any. Single-stranded RNA nucleotides carrying the putative binding 3’UTR seed regions and flanking nucleotides were immobilized in separate flow channels. Synthesized miR-181a at different concentrations was subjected in a single run. Kinetic binding constants were determined. Sensorgrams for the two flow channels containing the binding portions of the 3’UTR of CDKN1β and E2F7 shows that they both interacted significantly with miR-181a. As shown in Fig 2, the experimental curves of the binding between miR-181a and CDKN1β and E2F7 fit closely to a 1:1 binding model. The slight discrepancy between the theoretical and experimental curves is likely to be due to the aggregation of miR-181a at high concentrations. The dissociation constants (K_D_) for the binding between miR-181a and CDKN1β and E2F7 are 272.5 ± 0.008 nM and 1.186 ± 0.009 uM respectively, indicating that the binding between miR-181a and CDKN1β is stronger than that with E2F7, although a more extensive binding was predicted between miR-181a and E2F7.

The successful detection of interaction between miR-181a and the two seed regions (and the sites flanking them) of the 3’UTRs of CDKN1β and E2F7 mRNAs *in vitro* next led us to study if the interactions are also positive in the cellular environment.

### *In vivo* Binding Confirmation of miR-181a to the 3’UTR of CDKN1β and E2F7

**Fig 3:**
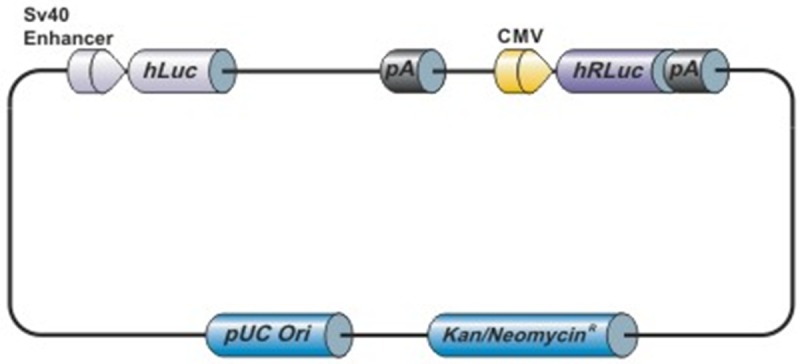
Schematic diagram of the firefly/renilla duo-luciferase reporter vector. The 3’UTRs of CDKN1β and E2F7 are cloned downstream of the firefly luciferase reporter gene, while a random sequence is cloned in the case of the control vector. Renilla luciferase activity is used as the internal control, and the firefly luciferase activity is normalized against that measured of renilla luciferase.

As the cellular environment is much different from that *in vitro*, in order to ensure that positive binding occurs between the miR-181a and the mRNAs in cells, we investigated the binding via a luciferase assay. Fig 3 shows the plasmid used. The plasmid is a firefly/Renilla Duo-Luciferase reporter vector, with the Renilla Luciferase reporter gene acting as the control reporter in which firefly luciferase is normalized against. Two different variations of the plasmids were used, corresponding to the different 3’UTRs of the mRNAs cloned downstream of the firefly luciferase reporter gene. An additional control vector (C1) was used that incorporated a random sequence downstream of the firefly luciferase reporter gene.

**Fig 4:**
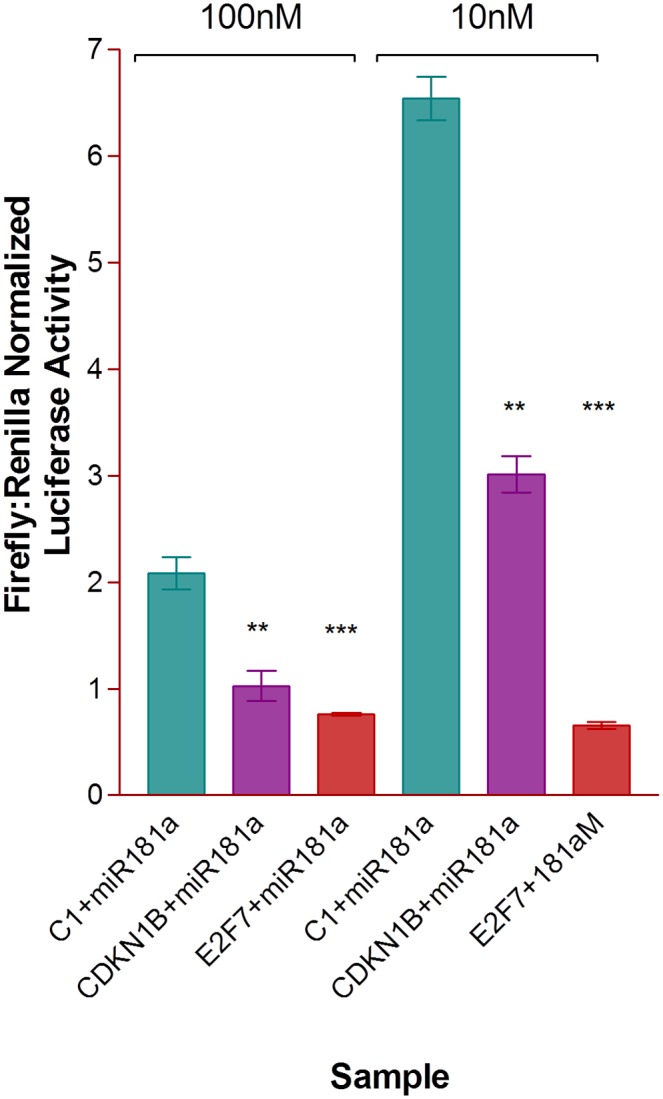
*In vivo* luciferase assay study of HepG2 cells co-transfected with the reporter plasmids and different concentrations of miR-181a. HepG2 cells were electroporated with 4ug of reporter plasmids and 10nM or 100nM miR-181a and seeded on 24 well plates. They were harvested after a 24h incubation and assayed for firefly and renilla luminescence using a manual luminometer. Results show that miR-181a binds to some extent, to the control vector, because of its dose dependent reduction in firefly luciferase activity upon transfection with the control vector. However, as compared to the control, miR-181a shows a significantly stronger binding to both the plasmids containing the 3’UTRs of CDKN1β and E2F7, with a further decrease in firefly luciferase activity by about two times when transfected with 10nM miR-181a and up to six times when transfected with 100nM miR-181a, as compared to when transfected with the control reporter plasmid.

Results (Fig 4) show that miR-181a binds to both the 3’UTRs of CDKN1β and E2F7 in HepG2 cells, confirming the previous findings of *in vitro* binding. A tenfold dilution of miR-181a was studied and in general, it seems that miR-181a does bind to the control plasmid to an extent, because the normalized firefly luciferase activity was lowered as the concentration of miR-181a increased from 10nM to 100nM. However, comparing data within the same miR-181a concentration shows that miR-181a binds to a larger extent to the 3’ UTRs of CDKN1β and E2F7 mRNAs as compared to the control plasmid, evident from the significant decrease in firefly luciferase activity detected. Not only that, but it seems that *in vivo*, miR-181a binds more strongly to the 3’ UTR of E2F7 than of CDKN1β mRNA. This is in contrast with the results of the *in vitro* SPR experiment, which demonstrated positive miR-181a binding to both seed region sequences of the two mRNAs, but more strongly towards that of CDKN1β instead of E2F7. It could be that the cellular environment is able to facilitate further, the binding of the miRNA to the 3’UTR of E2F7, due to the presence of various enzymes and RNA-induced silencing complex (RISC) that could aid strengthening the bond between miR-181a and the 3’UTR of E2F7. A more extensive binding between miR-181a and E2F7 was also previously predicted as compared that of CDKN1β by our bioinformatics analysis. In any case, miR-181a seems to target both mRNAs significantly, with p values = 0.0022 and 0.0008 for 100nM miR-181a targeting the 3’UTRs of CDKN1β and E2F7 respectively, and p values = 0.0057 and <0.0001 for 10nM miR-181a targeting the 3’UTRs of CDKN1β and E2F7 respectively.

### Western Blot Verification

**Fig 5:**
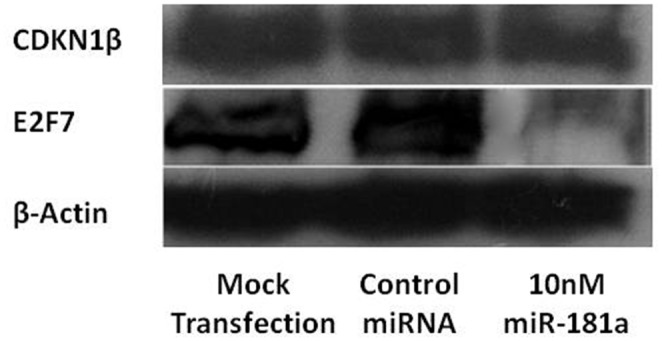
CDKN1β and E2F7 protein expression levels detection via a western blot analysis. HepG2 cells were starved, electroporated with 10nM miR-181a and harvested after 24h incubation. 30ug total protein extract was loaded onto each well of an SDS-PAGE and probed with various selected primary antibodies overnight at 4°C. MiR-181a reduced the protein expression levels of both CDKN1β and E2F7; with the reduction of E2F7 to a greater extent. β-actin served as a loading control.

**Fig 6:**
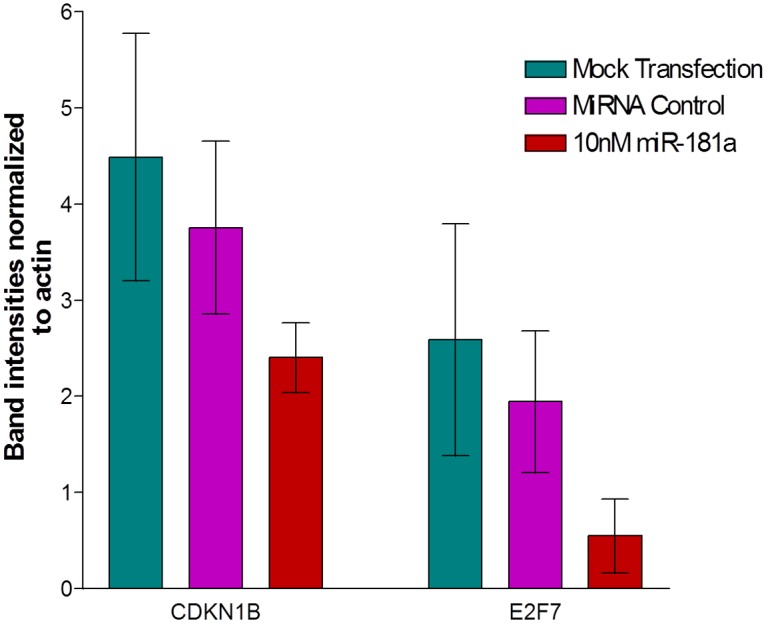
Protein levels of CDKN1β and E2F7in HepG2 cells following miR-181a transfection. Quantification of protein levels was analyzed using Image J software.

Binding of miR-181a to the 3’UTRs of CDKN1β and E2F7 *in vivo* may result in either the degradation of the mRNAs, the translational inhibition of the mRNAs to proteins or the temporary storage of the mRNAs in processing bodies (P-bodies) of cells and ultimately either degraded or released for delayed translation [11]. To study whether the protein levels of CDKN1β and E2F7 are affected by the transfection of miR-181a, we performed a western blot analysis. Figs 5 and 6 show that the transfection of 10nM miR-181a downregulates the protein levels of CDKN1β and E2F7. These two proteins were chosen in this study because our previous findings indicate that miR-181a, a miRNA found upregulated in HCC, causes a significant increase in HepG2 cell viability and may also play a part in cell cycle. These two proteins have been predicted by bioinformatics to be potential targets of miR-181a, and both partake in the negative regulation of the cell cycle and proliferation. CDKN1β is a well-known protein that prevents the activation of cyclin E-CDK2 and cyclin D-CDK4 complexes, thereby leading to a G_1_ cell cycle arrest [12]. E2F7 is a relatively newly discovered member of the E2F family of transcription factors that are highly involved in cell cycle progression, DNA repair and mitosis. In contrast to other well-studied E2F transcription factors (e.g. E2F1, E2F2 and E2F3), E2F7 acts in the cell cycle by being a transcriptional repressor and negatively regulates cell proliferation [13]. The downregulation of these two proteins by miR-181a could have been one of the reasons that led to the increase in HepG2 cell growth as seen in our previous study.

### MiR-181a Significantly Activates the MAPK/JNK Signaling Pathway in HepG2 Cells

**Fig 7:**
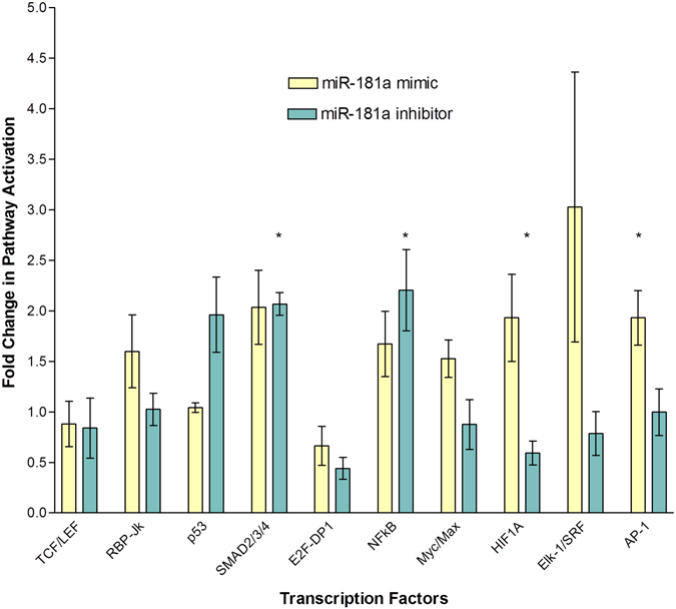
Microarray analysis of the expression levels/activities of ten important cancer-related transcription factors. HepG2 cells were reverse co-transfected with 100nM miR-181a or 100nM miR-181a inhibitor, along with the dried down transcription factor reporter plasmids and incubated for 24h in 96-well plates. Cells were assayed for luciferase activity 48h after reverse transfection using a plate luminometer. The values plotted are that of firefly luminescence normalized against that of renilla, which was used as the internal control. Among the ten pathways investigated, miR-181a caused a significant activation in the MAPK/JNK pathway, as seen by the increase in expression levels/activity of AP-1 protein. Inhibiting miR-181a significantly activated the NFκB and TGF-β pathways and lowered the HIF protein expression level/activity.

The identification of direct targets of miR-181a led us to further investigate the overall molecular effects it has in HepG2 cells. In order to do so, we carried out an analysis on important cancer pathways using the Cancer 10-pathway Reporter Luciferase Kit (Qiagen). Ten important cancer-related pathways can be studied using this assay simultaneously. The signaling pathways included are Wnt. Notch, p53/DNA damage, TGFβ, Cell cycle, NFκB, Myc/Max, Hypoxia, MAPK/ERK and MAPK/JNK. Positive and negative controls were also included for quality control purposes. The assay came in a 96-well plate format, with each column being distinct from the next, distinguishable by the different transcription factor reporters dried down in each well. HepG2 cells were reverse co-transfected with the transcription factor reporters and various miRNAs and subsequently assayed for luciferase activity. The normalized firefly:Renilla luciferase activity was then plotted for three independent experimental repeats. Fig 7 shows the effects of the transfection of miR-181a and its inhibitor in the various cancer-related signaling pathways, normalized with readouts obtained from the transfection of HepG2 cells with a control miRNA. Among the ten pathways analyzed, miR-181a caused the activation of activating protein 1 (AP-1) transcription factor most significantly (p value = 0.029), by approximately two folds, while inhibiting it abolished this observation. AP-1 is a transcriptional factor involved in the mitogen-activated protein (MAP) kinase signaling pathway. It contains two components, c-Jun and c-Fos, which are both crucial regulators of liver tumor development. C-Jun has been shown to promote the growth of tumor through the positive regulation of cell-cycle (e.g. Cyclin D1) or through the repression of its negative regulators (e.g. p16) [14]. It is also able to antagonize apoptosis in liver tumors. In addition to the increase in activity or expression levels of AP-1, other transcriptional factors were also shown to be affected by miR-181a in HepG2 cells. Transcription factors involved in NFκB, Myc, Hypoxia and MAPK/ERK also showed an increase in activity, albeit of lowered significance. These are signaling pathways often found activated in cancers that lead to increased cell proliferation, angiogenesis and evasion of apoptosis. Overall, miR-181a appears to activate most oncogenetic pathways.

One interesting observation though, is that the transfection of miR-181a also leads to the activation of TGF-β and Notch pathways, albeit of lowered significance. This translates to an increase in SMAD2/3/4 and RBP-Jk transcriptional activities, which, has a cell cycle inhibition effect and growth arresting effect respectively [15–16]. It is the overall balance of these different reinforcing and contradicting pathways that leads to the overall phenotype of the cell. Indeed, our previous study showed that cell viability was significantly increased when HepG2 cells were transfected with miR-181a.

In contrast to the increase in hypoxia-inducible factor s (HIF) transcriptional activity by miR-181a, inhibiting it caused a corresponding significant decrease by approximately 40% (p value = 0.027). Hypoxia is a condition in cells where oxygen concentration becomes low, leading to a massive transcription of genes by HIF proteins involved in angiogenesis, metabolism, cell proliferation and survival [17]. This is often seen in the case of many cancers, where oxygen concentration within cancer cells drop due to the spurt of cancer cell growth and HIF protein is overexpressed to accommodate this increase in cell proliferation. The lowered activity or expression levels of HIF in HepG2 cells transfected with miR-181a inhibitor could potentially decrease cell viability due to the reduction of this protective layer. Apart from the decrease in HIF, inhibiting miR-181a also caused a significant increase in SMAD protein activity (p value = 0.0024). This protein is involved in TGF-β signaling, which can act as a tumor suppressor by causing a G_1_ cell cycle arrest [15]. Also, the increase and decrease in p53 and E2F protein activities respectively may further contribute to the lowered cell proliferation and viability of HepG2 cells when miR-181a is inhibited. Overall, inhibiting miR-181a appears to activate most tumor-suppressing pathways. This is supported again by our previous study, where inhibiting miR-181a caused a significant decrease in HepG2 cell viability. However, the simultaneous activation of the NFκB pathway (p value = 0.041), may negate some of the anti-proliferative effects of the other tumor suppressive pathways, due to the ability of the NFκB pathway to induce cancer-causing cellular alterations like increasing insensitivity to growth inhibition, self-sufficiency in growth signals, evasion of apoptosis immortalization, angiogenesis and metastasis [18]. This may limit the therapeutic effects of inhibiting miR-181a. A combination of miR-181a inhibitor with specific siRNAs targeting undesirable tumor-promoting proteins could potentially further increase the eradication of cancer cells.

### MiR-181a Causes an Activation of SMAD, NFκB, and MAPK Signaling Pathways, Possibly Through Increased Expression of BMPR2

We previously identified CDKN1β and E2F7 as targets of miR-181a in HepG2 cells. In order to identify a larger range of targets, we made use of a custom-made array—miR-181 Targets RT^2^ Profiler PCR Array (Qiagen). In this array analysis, the expression of 84 hsa-miR-181a-5p target genes was profiled. This panel of 84 genes includes currently known experimentally verified plus bioinformatically predicted target genes regulated by hsa-miR-181a-5p. Using real-time PCR, we analyze the expression of a focused panel of genes likely to be regulated by miR-181a with this array. HepG2 cells were treated with 100nM miR-181a or 100nM miR-181a inhibitor and incubated for 24h before harvesting for RNA extraction. Extracted RNA was reverse transcribed and allowed to undergo PCR, with SYBR Green used as the reporter dye due to its ability to bind to double stranded DNA and its high sensitivity. Each run was done in triplicate. S1 and S2 Tables show the fold changes and p values of the test samples (i.e. HepG2 cells transfected with miR-181a or miR-181a inhibitor) vs control samples (i.e. HepG2 cells transfected with control miRNA) for all the genes probed for in the array. P values in bold indicate significance (i.e. P value<0.05). Overall, we do see fold changes in most of the genes, both up and down regulated. The array is custom-made to probe for predicted genes targeted by miR-181a. Instead of downregulating most genes, we see a mix of both up and downregulation. This highly supports the general consensus that most human miRNAs affect gene expression at the translational level instead of causing mRNA degradation, since considerable amount of mRNA could still be detected. Not only that, but evidently from our previous transcription factor array study, the transfection of miR-181a causes a change in the expression levels or activities of transcription factors in HepG2 cells that are not known to be its direct targets. This in turn means that miRNAs are able to affect gene expression of mRNAs directly or indirectly, due to the vast interconnectedness of biological molecules in the cell. Therefore, in the analysis of this study, we broaden the scope of the term ‘targets’ to include not just the possible direct targets, but the overall gene expression of HepG2 cells due to the direct or indirect effects of miR-181a and its inhibitor.

We will discuss the effects of miR-181a and its inhibitor on genes that have shown significant up or down regulation. Only one gene showed a consistent and significant change in expression level when miR-181a was transfected into HepG2 cells. Bone morphogenetic protein receptor type 2 (BMPR2) mRNA expression level is shown to be significantly upregulated by a factor of 1.64 (p value = 0.02) as compared to the control sample. The protein expressed by this gene is a serine/threonine receptor kinase that binds to bone morphogenetic proteins (BMPs) that leads to the transduction of cell signals involved in the SMAD, MAPK, NFκB, LIM domain kinase 1 (LIMK) and dynein, light chain, Tctex-type 1 (TCTEX) and v-src sarcoma viral oncogene homolog (SRC) signaling pathway [19]. As found earlier in our previous array pathway analysis, SMAD2/3/4, NFκB, Elk-1/SRF and AP-1 transcription factors corresponding to SMAD, NFκB, and MAPK signaling pathways were found to be over-activated in HepG2 cells due to the transfection of miR-181a. The increased gene expression of BMPR2 due to miR-181a could have led to the overall activation of these pathways as its binding to BMPs aids in mediating these signal transductions.

When miR-181a was inhibited in HepG2 cells, three genes showed a significant change in expression. GATA6, NOTCH4 and ZNF180 mRNA were found to increase by 1.89, 1.30 and 1.53 times respectively (p values = 0.0438, 0.0200 and 0.0136). A study [6] has shown that miR-181a downregulates GATA6 protein in Hep3B cells and also binds to its mRNA. It also showed that the inhibition of miR-181a restored the expression of GATA6 protein. Our studies on HepG2 cells show that inhibiting miR-181a significantly increases the expression of GATA6 mRNA. GATA6 is one of the regulators of hepatic cell differentiation. It is also believed that the loss of expression of this gene leads to the ‘stemness’ of HpSC-HCC, thereby giving hepatic cancer stem cells (CSCs) the ability to proliferate indefinitely. The increased gene expression of GATA6 due to the inhibition of miR-181a may lead to a reduction in ‘stemness’ of HpSC-HCC, thereby making it more vulnerable to HCC therapy.

The inhibition of miR-181a caused a significant increase in NOTCH4 mRNA. While NOTCH1 is a relatively well-studied gene that when activated, causes apoptosis and inhibition of HCC proliferation, NOTCH4 is less understood. There are studies that have shown that NOTCH4 expression inhibits angiogenesis, endothelial sprouting and migration through collagen [20]. Other studies however, show that NOTCH4 is upregulated in neoplastic hepatocytes with respect to normal liver cells [21]. Its functional implication, however, is still not known. Similar to NOTCH4, ZNF180 is found to be significantly upregulated when miR-181a was inhibited in HepG2 cells from our array analysis. However, its role in HCC is not well understood. Its plays a role mainly in the physiological and pathological processes, but their mechanisms of actions are still not known [22].

## Conclusion

MiR-181a plays a part in HCC, but its mechanism of action has yet to be elucidated. In this study, we have shown that miR-181a binds to the 3’UTRs of CDKN1β and E2F7 in HepG2 cells, and led to a reduction in their protein expression levels. These two proteins negatively regulate the cell cycle. Therefore, their targeting and downregulation by miR-181a may potentially impact HepG2 cell proliferation. Our earlier cell viability assays support this result, as a significant increase in cell viability was observed when HepG2 cells were transfected with miR-181a. Inhibiting it, on the other hand, significantly decreased cell viability.

Apart from the impact on these two cell cycle genes, miR-181a is also found to affect important transcription factors involved in cancer. These transcription factors play a part in cellular growth, angiogenesis, apoptosis and metastasis. One pathway significantly activated by miR-181a is MAPK/JNK, as seen from the increased transcriptional activity of AP-1. MAPK/JNK activation has been shown to lead to proliferation and tumorigenesis. Other cancer-causing pathways activated by miR-181a include NFκB, Myc, HIF and MAPK/ERK, albeit to a lesser extent.

Inhibiting miR-181a leads to an inactivation of HIF as well as a simultaneous activation of SMAD2/3/4 pathways. HIF production is a classic sign of hypoxia, a condition where oxygen supply is low. This is a condition common in cancers due to their increased growth rate and metabolism. The decrease in HIF activity may lead to a decrease in overall cellular metabolism, therefore resulting in a reduction in cell viability. Furthermore, the activation of SMAD2/3/4 transcriptional factors is known to lead to a G_1_ cell cycle arrest, potentially causing a further decrease in cell viability. This phenotype was observed in our previous functional analysis, where HepG2 cell viability significantly decreased when miR-181a was inhibited.

**Fig 8:**
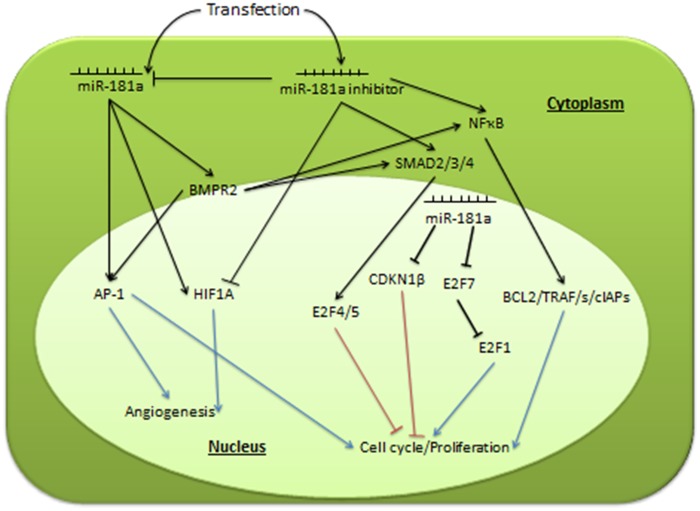
Illustration of possible pathways affected by miR-181a in HepG2 cells.

While miR-181a caused an activation of most tumor-promoting pathways, inhibiting it caused an activation of most tumor-suppressing pathways. In this study, we successfully identified CDKN1β and E2F7 as cellular cell cycle targets of miR-181a, and have also shown its effects on important pathways in cancer progression. It appears to have an overall oncogenetic effect, by activating more tumor-causing than tumor suppressing pathways, possibly through the increase in BMPR2 gene expression. Our findings open up a new approach in designing functional analysis of miRNAs in cellular processes, through the identification of their cellular targets. Fig 8 summarizes our findings and potential pathways affected by miR-181a in HepG2 cells.

## Supporting Information

S1 Table Fold change and p values of genes probed in HepG2 cells transfected with miR-181a. HepG2 cells were transfected with 100nM miR-181a, total RNA extracted and reverse transcribed. PCR was done on the cDNAs in the arrays using SYBR Green as the reporter dye. ΔCt represents Ct(Gene of interest)-AvgCt(housekeeping genes), while the fold change is represented by ΔΔCt = 2^(- Delta Ct)) in the Test Sample divided the normalized gene expression (2^(- Delta Ct)) in the Control Sample.
(DOCX)

S2 Table Fold change and p values of genes probed in HepG2 cells transfected with miR-181a inhibitor. HepG2 cells were transfected with 100nM miR-181a inhibitor, total RNA extracted and reverse transcribed. PCR was done on the cDNAs in the arrays using SYBR Green as the reporter dye. ΔCt represents Ct(Gene of interest)-AvgCt(housekeeping genes), while the fold change is represented by ΔΔCt = 2^(- Delta Ct)) in the Test Sample divided the normalized gene expression (2^(- Delta Ct)) in the Control Sample.
(DOCX)
