# Supplementary material for: Identification of Cellular Targets of MicroRNA-181a in HepG2 Cells: A New Approach for Functional Analysis of MicroRNAs
Source: PLoS One. 2015 Apr 22;10(4):e0123167. doi: 10.1371/journal.pone.0123167 (PMC4406611; doi:10.1371/journal.pone.0123167)
Supplement: S1 Table — HepG2 cells were transfected with 100nM miR-181a, total RNA extracted and reverse transcribed. PCR was done on the cDNAs in the arrays using SYBR Green as the reporter dye. ΔCt represents Ct(Gene of interest)-AvgCt(housekeeping genes), while the fold change is represented by ΔΔCt = 2^(- Delta Ct)) in the Test Sample divided the normalized gene expression (2^(- Delta Ct)) in the Control Sample. (DOCX) [file pone.0123167.s001.docx]

| **Symbol** | **Well** | **AVG ΔC_t_** | | **2^-ΔC_t_** | | **Fold** | **T-TEST** | **Fold Up- or** |
| --- | --- | --- | --- | --- | --- | --- | --- | --- |
|  |  | **(Ct(GOI) - Ave** | |  | | **Change** |  | **Down-** |
|  |  | **Ct (HKG))** | |  | |  |  | **Regulation** |
|  |  | **miR-** | **Control** | **miR-** | **Control** | **miR-181aM** | **p value** | **miR-181aM** |
|  |  | **181aM** | **Sample** | **181aM** | **Sample** | **/Control** |  | **/Control** |
| ACVR2A | A01 | 8.32 | 8.57 | 3.1E-03 | 2.6E-03 | 1.19 | 0.267537 | 1.19 |
| ADARB1 | A02 | 5.45 | 5.91 | 2.3E-02 | 1.7E-02 | 1.37 | 0.457283 | 1.37 |
| ADCY1 | A03 | 19.40 | 18.91 | 1.4E-06 | 2.0E-06 | 0.71 | 0.456841 | -1.41 |
| AICDA | A04 | 17.22 | 19.77 | 6.6E-06 | 1.1E-06 | **5.87** | 0.182613 | **5.87** |
| ATG5 | A05 | 7.25 | 7.55 | 6.6E-03 | 5.3E-03 | 1.23 | 0.498072 | 1.23 |
| ATM | A06 | 10.39 | 10.94 | 7.5E-04 | 5.1E-04 | 1.47 | 0.291322 | 1.47 |
| BCL2 | A07 | 10.40 | 10.80 | 7.4E-04 | 5.6E-04 | 1.32 | 0.165535 | 1.32 |
| BCL2L11 | A08 | 8.94 | 8.80 | 2.0E-03 | 2.2E-03 | 0.91 | 0.438811 | -1.10 |
| BDNF | A09 | 9.03 | 9.38 | 1.9E-03 | 1.5E-03 | 1.27 | 0.207232 | 1.27 |
| BMPR2 | A10 | 8.16 | 8.87 | 3.5E-03 | 2.1E-03 | 1.64 | **0.019618** | 1.64 |
| C16orf87 | A11 | 6.46 | 6.36 | 1.1E-02 | 1.2E-02 | 0.93 | 0.659065 | -1.07 |
| C6orf62 | A12 | 3.80 | 4.14 | 7.2E-02 | 5.7E-02 | 1.27 | 0.500807 | 1.27 |
| CAPRIN2 | B01 | 9.16 | 9.49 | 1.7E-03 | 1.4E-03 | 1.26 | 0.485955 | 1.26 |
| CASP3 | B02 | 7.47 | 5.95 | 5.7E-03 | 1.6E-02 | 0.35 | 0.313767 | -2.86 |
| CBLB | B03 | 8.42 | 9.09 | 2.9E-03 | 1.8E-03 | 1.59 | 0.402325 | 1.59 |
| CBX7 | B04 | 10.77 | 11.23 | 5.7E-04 | 4.2E-04 | 1.37 | 0.840424 | 1.37 |
| CD69 | B05 | 19.42 | 20.93 | 1.4E-06 | 5.0E-07 | 2.85 | 0.347530 | 2.85 |
| CDKN1B | B06 | 5.24 | 5.46 | 2.7E-02 | 2.3E-02 | 1.17 | 0.540722 | 1.17 |
| CDX2 | B07 | 17.64 | 14.34 | 4.9E-06 | 4.8E-05 | **0.10** | 0.373920 | **-9.86** |
| COPS2 | B08 | 4.56 | 4.59 | 4.2E-02 | 4.1E-02 | 1.03 | 0.983017 | 1.03 |
| CXCR3 | B09 | 15.80 | 17.23 | 1.7E-05 | 6.5E-06 | 2.69 | 0.091385 | 2.69 |
| CYLD | B10 | 7.70 | 7.79 | 4.8E-03 | 4.5E-03 | 1.06 | 0.122809 | 1.06 |
| DDIT4 | B11 | 6.84 | 6.31 | 8.7E-03 | 1.3E-02 | 0.69 | 0.443617 | -1.44 |
| DISC1 | B12 | 12.28 | 13.37 | 2.0E-04 | 9.4E-05 | 2.14 | 0.154479 | 2.14 |
| DOCK4 | C01 | 12.18 | 12.82 | 2.2E-04 | 1.4E-04 | 1.57 | 0.540829 | 1.57 |
| DUSP5 | C02 | 6.42 | 7.21 | 1.2E-02 | 6.7E-03 | 1.73 | 0.235421 | 1.73 |
| DUSP6 | C03 | 9.63 | 8.72 | 1.3E-03 | 2.4E-03 | 0.53 | 0.404475 | -1.88 |
| EIF4A2 | C04 | 2.87 | 2.95 | 1.4E-01 | 1.3E-01 | 1.06 | 0.949822 | 1.06 |
| ENKUR | C05 | 14.96 | 14.96 | 3.1E-05 | 3.1E-05 | 0.99 | 0.751036 | -1.01 |
| ETV6 | C06 | 7.53 | 8.11 | 5.4E-03 | 3.6E-03 | 1.49 | 0.140128 | 1.49 |
| FBXL3 | C07 | 5.32 | 3.73 | 2.5E-02 | 7.5E-02 | 0.33 | 0.379105 | **-3.02** |
| FKBP1A | C08 | 5.50 | 5.11 | 2.2E-02 | 2.9E-02 | 0.77 | 0.464486 | -1.31 |
| FOS | C09 | 8.19 | 8.37 | 3.4E-03 | 3.0E-03 | 1.13 | 0.829436 | 1.13 |
| GABRA1 | C10 | 19.32 | 20.93 | 1.5E-06 | 5.0E-07 | **3.05** | 0.367941 | **3.05** |
| GATA6 | C11 | 7.72 | 8.27 | 4.7E-03 | 3.2E-03 | 1.46 | 0.255139 | 1.46 |
| GLS | C12 | 4.69 | 5.04 | 3.9E-02 | 3.0E-02 | 1.27 | 0.331817 | 1.27 |
| GRIA1 | D01 | 20.78 | 17.44 | 5.5E-07 | 5.6E-06 | **0.10** | 0.373936 | **-10.13** |
| GRIA2 | D02 | 20.78 | 18.01 | 5.5E-07 | 3.8E-06 | **0.15** | 0.371367 | **-6.82** |
| GRIK1 | D03 | 12.89 | 13.84 | 1.3E-04 | 6.8E-05 | 1.93 | 0.071683 | 1.93 |
| HIPK2 | D04 | 14.19 | 14.04 | 5.3E-05 | 5.9E-05 | 0.90 | 0.833027 | -1.11 |
| HK2 | D05 | 4.69 | 4.93 | 3.9E-02 | 3.3E-02 | 1.18 | 0.486988 | 1.18 |
| HMGB2 | D06 | 4.07 | 4.30 | 5.9E-02 | 5.1E-02 | 1.17 | 0.392232 | 1.17 |
| IGF1R | D07 | 6.77 | 6.17 | 9.2E-03 | 1.4E-02 | 0.66 | 0.444346 | -1.51 |
| IL1A | D08 | 8.54 | 8.80 | 2.7E-03 | 2.2E-03 | 1.20 | 0.922057 | 1.20 |
| KANK1 | D09 | 5.95 | 5.18 | 1.6E-02 | 2.7E-02 | 0.59 | 0.336143 | -1.70 |
| KAT2B | D10 | 8.75 | 8.71 | 2.3E-03 | 2.4E-03 | 0.97 | 0.980091 | -1.03 |
| KCNA4 | D11 | 18.40 | 18.23 | 2.9E-06 | 3.3E-06 | 0.89 | 0.820454 | -1.12 |
| KIAA0195 | D12 | 8.08 | 8.53 | 3.7E-03 | 2.7E-03 | 1.36 | 0.341902 | 1.36 |
| KLHL2 | E01 | 8.15 | 8.27 | 3.5E-03 | 3.2E-03 | 1.09 | 0.589740 | 1.09 |
| KRAS | E02 | 6.13 | 6.40 | 1.4E-02 | 1.2E-02 | 1.20 | 0.674682 | 1.20 |
| LRBA | E03 | 7.65 | 7.98 | 5.0E-03 | 4.0E-03 | 1.26 | 0.447922 | 1.26 |
| MAP1B | E04 | 9.48 | 9.75 | 1.4E-03 | 1.2E-03 | 1.21 | 0.627909 | 1.21 |
| MAP3K10 | E05 | 8.14 | 8.83 | 3.5E-03 | 2.2E-03 | 1.62 | 0.269571 | 1.62 |
| MGMT | E06 | 6.45 | 7.67 | 1.1E-02 | 4.9E-03 | 2.34 | 0.374145 | 2.34 |
| NARF | E07 | 6.59 | 6.29 | 1.0E-02 | 1.3E-02 | 0.81 | 0.348006 | -1.23 |
| NLK | E08 | 7.80 | 8.08 | 4.5E-03 | 3.7E-03 | 1.21 | 0.654475 | 1.21 |
| NMT2 | E09 | 8.20 | 7.67 | 3.4E-03 | 4.9E-03 | 0.69 | 0.323736 | -1.45 |
| NOTCH4 | E10 | 13.23 | 13.69 | 1.0E-04 | 7.5E-05 | 1.38 | 0.280996 | 1.38 |
| NPEPPS | E11 | 5.88 | 5.96 | 1.7E-02 | 1.6E-02 | 1.06 | 0.849837 | 1.06 |
| PLAG1 | E12 | 16.85 | 15.56 | 8.5E-06 | 2.1E-05 | 0.41 | 0.158431 | -2.44 |
| PLAU | F01 | 16.76 | 18.32 | 9.0E-06 | 3.1E-06 | 2.94 | 0.277788 | 2.94 |
| PLCL2 | F02 | 6.90 | 5.96 | 8.4E-03 | 1.6E-02 | 0.52 | 0.320174 | -1.91 |
| PRKCD | F03 | 8.56 | 9.75 | 2.7E-03 | 1.2E-03 | 2.29 | 0.060598 | 2.29 |
| PROX1 | F04 | 20.78 | 18.99 | 5.5E-07 | 1.9E-06 | **0.29** | 0.374823 | **-3.46** |
| PTPN11 | F05 | 4.13 | 4.18 | 5.7E-02 | 5.5E-02 | 1.04 | 0.656689 | 1.04 |
| PTPN22 | F06 | 14.13 | 10.42 | 5.6E-05 | 7.3E-04 | **0.08** | 0.373865 | **-13.05** |
| RALA | F07 | 5.20 | 5.36 | 2.7E-02 | 2.4E-02 | 1.12 | 0.634097 | 1.12 |
| RLF | F08 | 7.30 | 7.47 | 6.3E-03 | 5.6E-03 | 1.13 | 0.156487 | 1.13 |
| RNF2 | F09 | 6.51 | 6.85 | 1.1E-02 | 8.7E-03 | 1.26 | 0.495303 | 1.26 |
| SIRT1 | F10 | 6.76 | 7.00 | 9.3E-03 | 7.8E-03 | 1.18 | 0.701498 | 1.18 |
| SLC2A1 | F11 | 4.62 | 4.75 | 4.1E-02 | 3.7E-02 | 1.09 | 0.436090 | 1.09 |
| STAT1 | F12 | 2.73 | 4.64 | 1.5E-01 | 4.0E-02 | **3.75** | 0.169613 | **3.75** |
| TANC2 | G01 | 6.98 | 7.29 | 7.9E-03 | 6.4E-03 | 1.23 | 0.940822 | 1.23 |
| TBPL1 | G02 | 7.90 | 7.80 | 4.2E-03 | 4.5E-03 | 0.93 | 0.820814 | -1.08 |
| TCERG1 | G03 | 4.73 | 4.20 | 3.8E-02 | 5.4E-02 | 0.69 | 0.535745 | -1.44 |
| TCL1A | G04 | 16.76 | 15.85 | 9.0E-06 | 1.7E-05 | 0.53 | 0.693015 | -1.88 |
| TMEM131 | G05 | 6.44 | 6.78 | 1.1E-02 | 9.1E-03 | 1.26 | 0.318019 | 1.26 |
| VSNL1 | G06 | 15.20 | 20.03 | 2.7E-05 | 9.4E-07 | **28.34** | 0.128277 | **28.34** |
| YTHDC1 | G07 | 5.76 | 5.91 | 1.8E-02 | 1.7E-02 | 1.11 | 0.808541 | 1.11 |
| ZFP36L1 | G08 | 4.34 | 3.76 | 4.9E-02 | 7.4E-02 | 0.67 | 0.409457 | -1.50 |
| ZFP36L2 | G09 | 4.68 | 4.22 | 3.9E-02 | 5.4E-02 | 0.73 | 0.461535 | -1.38 |
| ZNF180 | G10 | 7.79 | 8.10 | 4.5E-03 | 3.7E-03 | 1.23 | 0.257589 | 1.23 |
| ZNF37A | G11 | 6.66 | 7.38 | 9.9E-03 | 6.0E-03 | 1.65 | 0.248678 | 1.65 |
| ZNF83 | G12 | 12.00 | 12.12 | 2.4E-04 | 2.2E-04 | 1.09 | 0.778311 | 1.09 |
| ACTB | H01 | -1.68 | -2.17 | 3.2E+00 | 4.5E+00 | 0.71 | 0.280654 | -1.41 |
| B2M | H02 | -0.21 | 0.02 | 1.2E+00 | 9.9E-01 | 1.17 | 0.634824 | 1.17 |
| GAPDH | H03 | -1.27 | -0.92 | 2.4E+00 | 1.9E+00 | 1.28 | 0.722400 | 1.28 |
| HPRT1 | H04 | 4.23 | 3.78 | 5.3E-02 | 7.3E-02 | 0.73 | 0.468227 | -1.36 |
| RPLP0 | H05 | -1.07 | -0.71 | 2.1E+00 | 1.6E+00 | 1.28 | 0.087210 | 1.28 |
