# Supplementary material for: Identification of Cellular Targets of MicroRNA-181a in HepG2 Cells: A New Approach for Functional Analysis of MicroRNAs
Source: PLoS One. 2015 Apr 22;10(4):e0123167. doi: 10.1371/journal.pone.0123167 (PMC4406611; doi:10.1371/journal.pone.0123167)
Supplement: S2 Table — HepG2 cells were transfected with 100nM miR-181a inhibitor, total RNA extracted and reverse transcribed. PCR was done on the cDNAs in the arrays using SYBR Green as the reporter dye. ΔCt represents Ct(Gene of interest)-AvgCt(housekeeping genes), while the fold change is represented by ΔΔCt = 2^(- Delta Ct)) in the Test Sample divided the normalized gene expression (2^(- Delta Ct)) in the Control Sample. (DOCX) [file pone.0123167.s002.docx]

| **Symbol** | **Well** | **AVG ΔC_t_** | | **2^-ΔC_t_** | | **Fold** | **T-TEST** | **Fold Up- or** |
| --- | --- | --- | --- | --- | --- | --- | --- | --- |
|  |  | **(Ct(GOI) - Ave Ct** | |  | | **Change** |  | **Down-** |
|  |  | **(HKG))** | |  | |  |  | **Regulation** |
|  |  | **miR-** | **Control** | **miR-** | **Control** | **miR-181a** | **p value** | **miR-181a** |
|  |  | **181a** | **Sample** | **181a** | **Sample** | **inhibitor** |  | **inhibitor** |
|  |  | **inhibitor** |  | **inhibitor** |  | **/Control** |  | **/Control** |
| ACVR2A | A01 | 8.16 | 8.57 | 3.5E-03 | 2.6E-03 | 1.33 | 0.178884 | 1.33 |
| ADARB1 | A02 | 5.62 | 5.91 | 2.0E-02 | 1.7E-02 | 1.22 | 0.681159 | 1.22 |
| ADCY1 | A03 | 19.15 | 18.91 | 1.7E-06 | 2.0E-06 | 0.85 | 0.927969 | -1.18 |
| AICDA | A04 | 19.09 | 19.77 | 1.8E-06 | 1.1E-06 | 1.60 | 0.575954 | 1.60 |
| ATG5 | A05 | 7.43 | 7.55 | 5.8E-03 | 5.3E-03 | 1.09 | 0.881300 | 1.09 |
| ATM | A06 | 10.52 | 10.94 | 6.8E-04 | 5.1E-04 | 1.34 | 0.432982 | 1.34 |
| BCL2 | A07 | 10.47 | 10.80 | 7.0E-04 | 5.6E-04 | 1.25 | 0.191383 | 1.25 |
| BCL2L11 | A08 | 8.81 | 8.80 | 2.2E-03 | 2.2E-03 | 1.00 | 0.949859 | -1.00 |
| BDNF | A09 | 9.41 | 9.38 | 1.5E-03 | 1.5E-03 | 0.98 | 0.883756 | -1.02 |
| BMPR2 | A10 | 8.14 | 8.87 | 3.6E-03 | 2.1E-03 | 1.66 | 0.261549 | 1.66 |
| C16orf87 | A11 | 6.73 | 6.36 | 9.4E-03 | 1.2E-02 | 0.77 | 0.488151 | -1.30 |
| C6orf62 | A12 | 4.13 | 4.14 | 5.7E-02 | 5.7E-02 | 1.00 | 0.869187 | 1.00 |
| CAPRIN2 | B01 | 9.43 | 9.49 | 1.5E-03 | 1.4E-03 | 1.05 | 0.822617 | 1.05 |
| CASP3 | B02 | 7.70 | 5.95 | 4.8E-03 | 1.6E-02 | **0.30** | 0.294857 | **-3.36** |
| CBLB | B03 | 8.53 | 9.09 | 2.7E-03 | 1.8E-03 | 1.47 | 0.532407 | 1.47 |
| CBX7 | B04 | 9.99 | 11.23 | 9.8E-04 | 4.2E-04 | 2.35 | 0.185352 | 2.35 |
| CD69 | B05 | 19.39 | 20.93 | 1.5E-06 | 5.0E-07 | 2.91 | 0.323067 | 2.91 |
| CDKN1B | B06 | 5.77 | 5.46 | 1.8E-02 | 2.3E-02 | 0.81 | 0.430697 | -1.24 |
| CDX2 | B07 | 18.56 | 14.34 | 2.6E-06 | 4.8E-05 | **0.05** | 0.373911 | **-18.68** |
| COPS2 | B08 | 4.75 | 4.59 | 3.7E-02 | 4.1E-02 | 0.90 | 0.658805 | -1.11 |
| CXCR3 | B09 | 17.18 | 17.23 | 6.7E-06 | 6.5E-06 | 1.04 | 0.792632 | 1.04 |
| CYLD | B10 | 7.73 | 7.79 | 4.7E-03 | 4.5E-03 | 1.04 | 0.630303 | 1.04 |
| DDIT4 | B11 | 7.21 | 6.31 | 6.8E-03 | 1.3E-02 | 0.54 | 0.405868 | -1.86 |
| DISC1 | B12 | 12.76 | 13.37 | 1.4E-04 | 9.4E-05 | 1.53 | 0.256660 | 1.53 |
| DOCK4 | C01 | 12.71 | 12.82 | 1.5E-04 | 1.4E-04 | 1.08 | 0.775658 | 1.08 |
| DUSP5 | C02 | 6.66 | 7.21 | 9.9E-03 | 6.7E-03 | 1.47 | 0.468794 | 1.47 |
| DUSP6 | C03 | 9.91 | 8.72 | 1.0E-03 | 2.4E-03 | 0.44 | 0.368368 | -2.28 |
| EIF4A2 | C04 | 3.65 | 2.95 | 7.9E-02 | 1.3E-01 | 0.62 | 0.192413 | -1.62 |
| ENKUR | C05 | 14.45 | 14.96 | 4.5E-05 | 3.1E-05 | 1.42 | 0.480452 | 1.42 |
| ETV6 | C06 | 7.78 | 8.11 | 4.6E-03 | 3.6E-03 | 1.26 | 0.294626 | 1.26 |
| FBXL3 | C07 | 4.82 | 3.73 | 3.5E-02 | 7.5E-02 | 0.47 | 0.417681 | -2.13 |
| FKBP1A | C08 | 5.36 | 5.11 | 2.4E-02 | 2.9E-02 | 0.84 | 0.570747 | -1.19 |
| FOS | C09 | 8.56 | 8.37 | 2.6E-03 | 3.0E-03 | 0.88 | 0.664234 | -1.14 |
| GABRA1 | C10 | 19.23 | 20.93 | 1.6E-06 | 5.0E-07 | **3.26** | 0.309785 | **3.26** |
| GATA6 | C11 | 7.35 | 8.27 | 6.1E-03 | 3.2E-03 | 1.89 | **0.043807** | 1.89 |
| GLS | C12 | 4.46 | 5.04 | 4.5E-02 | 3.0E-02 | 1.49 | 0.092393 | 1.49 |
| GRIA1 | D01 | 19.43 | 17.44 | 1.4E-06 | 5.6E-06 | **0.25** | 0.377106 | **-3.95** |
| GRIA2 | D02 | 17.50 | 18.01 | 5.4E-06 | 3.8E-06 | 1.43 | 0.502535 | 1.43 |
| GRIK1 | D03 | 13.46 | 13.84 | 8.9E-05 | 6.8E-05 | 1.31 | 0.488462 | 1.31 |
| HIPK2 | D04 | 13.78 | 14.04 | 7.1E-05 | 5.9E-05 | 1.20 | 0.620013 | 1.20 |
| HK2 | D05 | 4.61 | 4.93 | 4.1E-02 | 3.3E-02 | 1.25 | 0.426343 | 1.25 |
| HMGB2 | D06 | 4.16 | 4.30 | 5.6E-02 | 5.1E-02 | 1.10 | 0.548619 | 1.10 |
| IGF1R | D07 | 6.95 | 6.17 | 8.1E-03 | 1.4E-02 | 0.58 | 0.410657 | -1.72 |
| IL1A | D08 | 9.57 | 8.80 | 1.3E-03 | 2.2E-03 | 0.59 | 0.539420 | -1.71 |
| KANK1 | D09 | 5.86 | 5.18 | 1.7E-02 | 2.7E-02 | 0.63 | 0.364501 | -1.59 |
| KAT2B | D10 | 8.53 | 8.71 | 2.7E-03 | 2.4E-03 | 1.13 | 0.603511 | 1.13 |
| KCNA4 | D11 | 18.16 | 18.23 | 3.4E-06 | 3.3E-06 | 1.05 | 0.718505 | 1.05 |
| KIAA0195 | D12 | 7.95 | 8.53 | 4.1E-03 | 2.7E-03 | 1.49 | 0.212327 | 1.49 |
| KLHL2 | E01 | 8.06 | 8.27 | 3.7E-03 | 3.2E-03 | 1.15 | 0.434422 | 1.15 |
| KRAS | E02 | 6.42 | 6.40 | 1.2E-02 | 1.2E-02 | 0.98 | 0.841916 | -1.02 |
| LRBA | E03 | 7.80 | 7.98 | 4.5E-03 | 4.0E-03 | 1.13 | 0.608517 | 1.13 |
| MAP1B | E04 | 9.48 | 9.75 | 1.4E-03 | 1.2E-03 | 1.21 | 0.630339 | 1.21 |
| MAP3K10 | E05 | 8.09 | 8.83 | 3.7E-03 | 2.2E-03 | 1.67 | 0.189699 | 1.67 |
| MGMT | E06 | 7.58 | 7.67 | 5.2E-03 | 4.9E-03 | 1.07 | 0.638796 | 1.07 |
| NARF | E07 | 6.40 | 6.29 | 1.2E-02 | 1.3E-02 | 0.93 | 0.605548 | -1.08 |
| NLK | E08 | 7.63 | 8.08 | 5.1E-03 | 3.7E-03 | 1.36 | 0.367233 | 1.36 |
| NMT2 | E09 | 8.20 | 7.67 | 3.4E-03 | 4.9E-03 | 0.69 | 0.326794 | -1.45 |
| NOTCH4 | E10 | 13.32 | 13.69 | 9.8E-05 | 7.5E-05 | 1.30 | **0.020019** | 1.30 |
| NPEPPS | E11 | 5.75 | 5.96 | 1.9E-02 | 1.6E-02 | 1.16 | 0.572568 | 1.16 |
| PLAG1 | E12 | 17.56 | 15.56 | 5.2E-06 | 2.1E-05 | **0.25** | 0.142215 | **-3.98** |
| PLAU | F01 | 16.33 | 18.32 | 1.2E-05 | 3.1E-06 | **3.97** | 0.237507 | **3.97** |
| PLCL2 | F02 | 6.46 | 5.96 | 1.1E-02 | 1.6E-02 | 0.71 | 0.561881 | -1.41 |
| PRKCD | F03 | 8.67 | 9.75 | 2.4E-03 | 1.2E-03 | 2.11 | 0.219765 | 2.11 |
| PROX1 | F04 | 20.49 | 18.99 | 6.8E-07 | 1.9E-06 | 0.35 | 0.383044 | -2.82 |
| PTPN11 | F05 | 4.48 | 4.18 | 4.5E-02 | 5.5E-02 | 0.81 | 0.286405 | -1.23 |
| PTPN22 | F06 | 15.65 | 10.42 | 1.9E-05 | 7.3E-04 | **0.03** | 0.373580 | **-37.54** |
| RALA | F07 | 5.00 | 5.36 | 3.1E-02 | 2.4E-02 | 1.28 | 0.219388 | 1.28 |
| RLF | F08 | 7.14 | 7.47 | 7.1E-03 | 5.6E-03 | 1.26 | 0.183147 | 1.26 |
| RNF2 | F09 | 6.38 | 6.85 | 1.2E-02 | 8.7E-03 | 1.39 | 0.250751 | 1.39 |
| SIRT1 | F10 | 6.78 | 7.00 | 9.1E-03 | 7.8E-03 | 1.16 | 0.762576 | 1.16 |
| SLC2A1 | F11 | 4.70 | 4.75 | 3.8E-02 | 3.7E-02 | 1.03 | 0.595572 | 1.03 |
| STAT1 | F12 | 3.24 | 4.64 | 1.1E-01 | 4.0E-02 | 2.64 | 0.424496 | 2.64 |
| TANC2 | G01 | 7.04 | 7.29 | 7.6E-03 | 6.4E-03 | 1.19 | 0.977385 | 1.19 |
| TBPL1 | G02 | 7.45 | 7.80 | 5.7E-03 | 4.5E-03 | 1.27 | 0.451731 | 1.27 |
| TCERG1 | G03 | 3.96 | 4.20 | 6.4E-02 | 5.4E-02 | 1.18 | 0.898740 | 1.18 |
| TCL1A | G04 | 18.44 | 15.85 | 2.8E-06 | 1.7E-05 | **0.17** | 0.209868 | **-6.04** |
| TMEM131 | G05 | 6.65 | 6.78 | 1.0E-02 | 9.1E-03 | 1.09 | 0.640466 | 1.09 |
| VSNL1 | G06 | 17.34 | 20.03 | 6.0E-06 | 9.4E-07 | **6.45** | 0.226328 | **6.45** |
| YTHDC1 | G07 | 5.73 | 5.91 | 1.9E-02 | 1.7E-02 | 1.13 | 0.826471 | 1.13 |
| ZFP36L1 | G08 | 4.36 | 3.76 | 4.9E-02 | 7.4E-02 | 0.66 | 0.400925 | -1.52 |
| ZFP36L2 | G09 | 4.34 | 4.22 | 4.9E-02 | 5.4E-02 | 0.92 | 0.646202 | -1.08 |
| ZNF180 | G10 | 7.48 | 8.10 | 5.6E-03 | 3.7E-03 | 1.53 | **0.013637** | 1.53 |
| ZNF37A | G11 | 6.39 | 7.38 | 1.2E-02 | 6.0E-03 | 1.98 | 0.068227 | 1.98 |
| ZNF83 | G12 | 12.77 | 12.12 | 1.4E-04 | 2.2E-04 | 0.64 | 0.328636 | -1.57 |
| ACTB | H01 | -1.79 | -2.17 | 3.5E+00 | 4.5E+00 | 0.77 | 0.405856 | -1.31 |
| B2M | H02 | -0.20 | 0.02 | 1.1E+00 | 9.9E-01 | 1.16 | 0.652129 | 1.16 |
| GAPDH | H03 | -1.23 | -0.92 | 2.3E+00 | 1.9E+00 | 1.24 | 0.796353 | 1.24 |
| HPRT1 | H04 | 4.14 | 3.78 | 5.7E-02 | 7.3E-02 | 0.78 | 0.484904 | -1.28 |
| RPLP0 | H05 | -0.93 | -0.71 | 1.9E+00 | 1.6E+00 | 1.16 | 0.418096 | 1.16 |
